# Modeling Host-Pathogen Interaction to Elucidate the Metabolic Drug Response of Intracellular *Mycobacterium tuberculosis*

**DOI:** 10.3389/fcimb.2019.00144

**Published:** 2019-05-08

**Authors:** Rienk A. Rienksma, Peter J. Schaap, Vitor A. P. Martins dos Santos, Maria Suarez-Diez

**Affiliations:** ^1^Laboratory of Systems and Synthetic Biology, Department of Agrotechnology and Food Sciences, Wageningen University & Research, Wageningen, Netherlands; ^2^LifeGlimmer GmbH, Berlin, Germany

**Keywords:** metabolic model, host-pathogen interaction, flux balance analysis, drug response, antibiotics, *Mycobacterium tuberculosis*

## Abstract

Little is known about the metabolic state of *Mycobacterium tuberculosis* (Mtb) inside the phagosome, a compartment inside phagocytes for killing pathogens and other foreign substances. We have developed a combined model of Mtb and human metabolism, sMtb-RECON and used this model to predict the metabolic state of Mtb during infection of the host. Amino acids are predicted to be used for energy production as well as biomass formation. Subsequently we assessed the effect of increasing dosages of drugs targeting metabolism on the metabolic state of the pathogen and predict resulting metabolic adaptations and flux rerouting through various pathways. In particular, the TCA cycle becomes more important upon drug application, as well as alanine, aspartate, glutamate, proline, arginine and porphyrin metabolism, while glycine, serine, and threonine metabolism become less important. We modeled the effect of 11 metabolically active drugs. Notably, the effect of eight could be recreated and two major profiles of the metabolic state were predicted. The profiles of the metabolic states of Mtb affected by the drugs BTZ043, cycloserine and its derivative terizidone, ethambutol, ethionamide, propionamide, and isoniazid were very similar, while TMC207 is predicted to have quite a different effect on metabolism as it inhibits ATP synthase and therefore indirectly interferes with a multitude of metabolic pathways.

## Introduction

*Mycobacterium tuberculosis* (Mtb), the etiological agent of tuberculosis, is an intracellular pathogen that thrives inside the phagosome of the host's macrophages (Gengenbacher and Kaufmann, 2012; Zondervan et al., 2018). This environment prevents obtaining accurate *in vivo* measurements characterizing the metabolic state of the pathogen during infection. Genome-scale models (GEM) of metabolism have been proposed as efficient tools to explore bacterial metabolism, even in conditions difficult to access experimentally. Flux balance analysis (FBA) is a widely used approach to study GEMs and relies on the definition of an objective function that characterizes the metabolic objective of the organism under study (Orth et al., 2010). Predictions made using GEMs are highly dependent on the objective that is being used and the constraints placed on the uptake and excretion of nutrients and metabolites. To perform predictions on *in vitro* growth, most often a biomass reaction is selected as objective function for maximization. The biomass reaction details the biomass composition in terms of its constituents such as proteins, lipids and nucleic acids. This composition might vary in different growth conditions (Hanegraaf and Muller, 2001). Biomass as an objective function has been used for the earliest genome-scale metabolic models of Mtb (Beste et al., 2007; Jamshidi and Palsson, 2007) as well as more recent models (Lofthouse et al., 2013; Rienksma et al., 2014; Vashisht et al., 2014; Garay et al., 2015; Ma et al., 2015; Puniya et al., 2016; Kavvas et al., 2018). Recently, a condition-specific biomass reaction has been formulated for Mtb inside the host by integrating model sMtb (Rienksma et al., 2014) and gene expression data during on-going infection (Rienksma et al., 2018).

*In silico* gene knockout analysis has been the method of choice to predict metabolic drug targets, in the form of genes and their associated enzyme products (Beste et al., 2007; Jamshidi and Palsson, 2007; Kavvas et al., 2018). Such methods are based on analyzing the effect of completely blocking the flux through the corresponding reactions on growth predictions. This full blockage approach would fail to predict cases wherein drugs reach Mtb in relatively small amounts so that enzyme function is only partly lost, allowing Mtb to counteract the negative effects of such a drug by altering its metabolic state to overcome non-optimal fluxes due to the drug affected enzymes. Bhat et al. (2010) developed a method to study dose dependent effects of isoniazid on the metabolic state that relied on simulating the effects of partial loss of function of the affected enzymes.

To capture the interaction between Mtb and its host on a metabolic level, a model of macrophage metabolism is required. Nutrients for Mtb are obtained from the phagosome, a cellular compartment specific to macrophages, and from the cytosol after Mtb gains access (Lerner et al., 2018). The phagosome represents a nutrient-poor, hypoxic, and nitrosative environment wherein Mtb is able to survive (Schnappinger et al., 2003). Nevertheless, the nutrients available in the phagosome, arguably after cytosolic access, are predicted to be varied (Beste et al., 2013; Zimmermann et al., 2017), and as such, allow metabolic flexibility of Mtb, which is best captured using a combined host-pathogen model. Although a host-pathogen metabolic model is more elaborate, and introduces more uncertainty and variability, several gene expression datasets have been introduced that cover both host and pathogen (Rienksma et al., 2015; Zimmermann et al., 2017), and are suited to constrain such a model to make it condition-specific.

For Mtb, a host-pathogen model was first created by Bordbar et al. (2010), based on iNJ661, a well-known Mtb model published in 2007 (Jamshidi and Palsson, 2007) and RECON 1, the first global human metabolic reconstruction (Duarte et al., 2007). The combined model allowed simulation of metabolic changes during infection and three distinct pathological states of Mtb were described.

Improved versions of the individual models describing the metabolism of host and pathogen are available. Model sMtb is a comprehensive model of Mtb metabolism with an increased scope of the underlying metabolic network and increased predictive power regarding the metabolic state and gene essentiality (Rienksma et al., 2014). RECON 2.2 (Thiele et al., 2013; Swainston et al., 2016), almost doubles the size of the metabolic network of RECON 1. Here, we integrate sMtb and RECON 2.2 to create an Mtb-Macrophage model, sMtb-RECON. The combined model has condition-specific objective functions for both pathogen and host, based on dual RNA-sequencing data. By applying various known metabolic drugs *in silico*, we highlight pathways that are important for Mtb to escape eradication by drug and host. Drugs that specifically target these pathways could therefore prove to be a valuable addition to the existing drugs.

## Materials and Methods

### Mtb and Human Models of Metabolism

We used the genome-scale metabolic model of *Mycobacterium tuberculosis* called sMtb, *in silico Mycobacterium tuberculosis* which represents a modification of the model presented in Rienksma et al. (2014). The GEM reconstruction of human metabolism RECON 2.2 (Swainston et al., 2016) was used as a model representing the host.

### Creating a Combined sMtb-RECON Model

**Figure 1:**
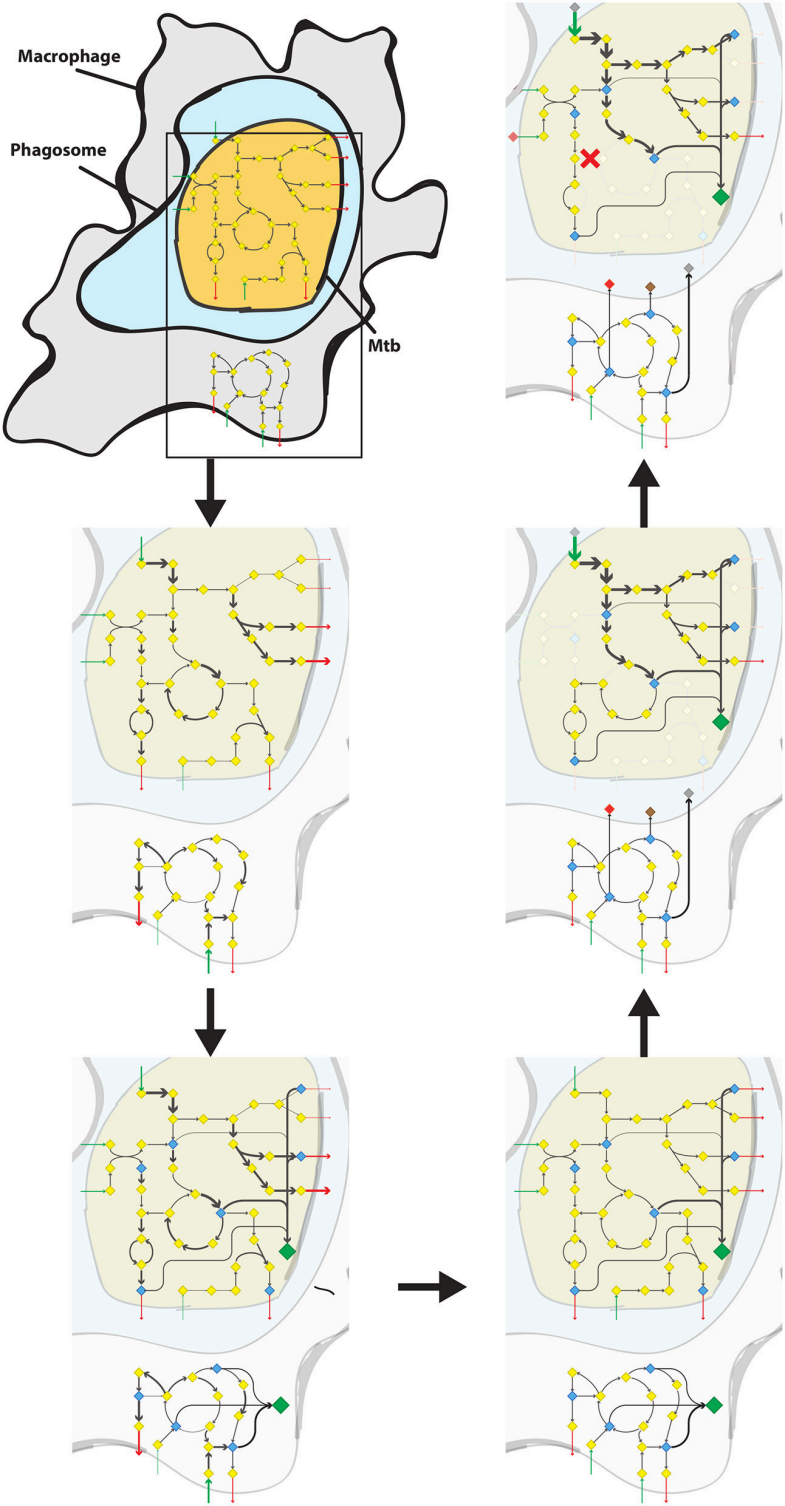
Predicting the metabolic state of Mtb during infection and drug application. Schematic overview of the steps required to calculate a metabolic state of Mtb during infection from the upper left panel following the arrows to the upper right panel. Mtb (yellow shape) is depicted inside the phagosome (light blue shape) of a macrophage (gray shape). Metabolisms of both Mtb and Macrophage are indicated with diamonds and arrows. Yellow diamonds represent metabolites, blue diamonds represent biomass precursors, gray/brown/red diamonds represent phagosomal nutrients, and the large green diamond represents the condition-specific biomass reaction. Gray arrows represent metabolic conversion rates, green arrows represent uptake rates, and red arrows represent secretion rates, wherein the thickness of the arrows is proportional to the rate. Red crosses represent blocked metabolic conversions. Upper left panel: A constraint-based genome-scale model of Mtb metabolism is coupled to one of human metabolism and a combined model is obtained. Middle left panel: Metabolic conversion rates (i.e., metabolic fluxes) are constrained proportional to mRNA transcript abundance in both Mtb and macrophage. Lower left panel: For each potential biomass precursor, the flux through metabolism toward that precursor is maximized, both for Mtb and the macrophage, obtaining two condition-specific biomass reactions. Lower right panel: The constraints based on mRNA transcript abundance are removed from the combined model. Middle right panel: The nutrients available for Mtb in the phagosome, and their maximum uptake rate are set according to the condition-specific biomass reaction of the macrophage. Subsequently, the condition-specific biomass reaction of Mtb is maximized, while the total flux through enzymatically-catalyzed reactions is minimized. Upper right panel: The rate of an enzymatically-catalyzed reaction is constricted by the effect of a metabolically active drug (red cross within the rounded square) and metabolic rerouting occurs toward a part of metabolism that contains a relatively higher number of enzymatically catalyzed reactions.

From the biomass precursors of the biomass reaction of RECON 2.2, all precursors were selected that could be present in the cytoplasm. As Mtb is known to be able to escape from the phagosome to the cytosol (van der Wel et al., 2007) and no phagosomal compartment was present in RECON 2.2 we took all metabolic precursors from the cytoplasm as biomass precursors for the macrophage condition-specific biomass reaction. A macrophage condition-specific biomass reaction was created using this list of biomass precursors and the gene expression profile of the macrophage-like THP-1 cells (Rienksma et al., 2015). The same method was applied to create an Mtb condition-specific biomass reaction using the gene expression profile of the Mtb-like *Mycobacterium bovis* BCG gene expression profile (Figure 1, up to the lower right panel). This condition-specific biomass reaction is used as a proxy for the number of available nutrients and their corresponding maximum uptake rates for Mtb in the combined sMtb-RECON model (Figure 1, middle right panel).

### Constraining the Combined Model With Gene Expression Data

Model sMtb and RECON 2.2 were constrained as described in Rienksma et al. (2018), using raw sequence read data available in the EMBL-EBI European Nucleotide Archive under the Accession No. PRJEB6552, http://www.ebi.ac.uk/ena/data/view/PRJEB6552 for *M. bovis* BCG cells infecting THP-1 cells. In brief, reads mapping to either *M. bovis* BCG genome or human transcriptome were used to constrain each model, respectively. In brief, reads aligning to each gene in the model were added. Genes with < 100 counts per million were considered lowly expressed and assigned a count value of zero. Gene count values were transferred to their corresponding reactions: for reactions catalyzed by isozymes, gene counts were added; for reactions catalyzed by a protein complex, the smallest number of counts associated to genes encoding a part of such a complex was assigned to the reaction; for reactions that can be catalyzed by different protein complexes, the smallest number of counts associated to genes encoding a part of a complex was assigned to each complex and the sum of the counts for all the complexes was assigned to the reaction. Total number of counts assigned to each reaction was normalized by dividing by the largest number of counts assigned to any reaction in the corresponding model, resulting in a value ranging between 0 and 1 for each enzyme-catalyzed reaction. Normalized values were used as upper (upper and lower) bounds on unidirectional (bidirectional) reactions.

### Obtaining a Condition-Specific Biomass Reaction for sMtb and a Phagosomal Environment

After assigning counts to the reactions in both sMtb and RECON 2.2 a condition-specific biomass reaction was obtained for sMtb as described in Rienksma et al. (2018). For RECON 2.2 a similar approach was taken. A list of cytoplasmic biomass precursors, obtained from the biomass reaction present in RECON 2.2 was used for maximization of said precursors one-by-one, while keeping all uptake rates for model RECON 2.2 unconstrained. The resulting condition-specific biomass reaction, which is in essence a ratio between different cytoplasmic biomass precursors, was used as a proxy for the phagosomal composition. These biomass precursor values were subsequently linearly scaled such that all values range between 0 and 1 mmol/h by dividing each value by the largest value obtained.

### Applying Constraints and Calculating a Reference Metabolic State

A reference metabolic state, representing non-drugged growth of Mtb in the phagosome, was calculated in a similar manner as previous efforts (Rienksma et al., 2018), using a bi-objective optimization method, with the exception of using the calculated phagosomal composition and its corresponding values as maximal allowable nutrient uptake rates. The ratio between biomass formation and enzyme usage is expressed by *f*_*r*_. There exists a range wherein biomass formation and enzyme usage are balanced. An *f*_*r*_ value of 0.8 was used for these calculations (Rienksma et al., 2018), wherein

$$f_{r}=\frac{f_{b}}{\sum_{i=1}^{n}f_{e,i}}$$

and *f*_*e, i*_ represents the weight factor for enzymatically catalyzed reaction *i* and *f*_*b*_ represents the weight factor for the biomass reaction.

### Creating a Weights Vector for Each Metabolic Drug

A list of available metabolic drugs was created and the genes encoding the enzymes that are known or expected to be affected by these drugs were listed (Table 1). For each drug, *i*, a vector, **c**_*d, i*_, containing weights ranging between 0 and 1 was created. A 0 represents a non-affected reaction and a 1 represents a fully affected reaction. A reaction that, for example, is catalyzed by three isozymes, and from among these isozymes only one is affected by the respective drug, would receive a value of 0.33. Likewise, if two out of the three isozymes are affected by the drug, a value of 0.67 is be attributed to that reaction. On the other hand, if the reaction would be catalyzed by a complex of three enzymes, a value of 1 would always be attributed to the respective reaction if at least one enzyme in the complex would be affected by the respective drug.

**Table 1:** Drugs acting on metabolic enzymes.

**Drug**	**Mode of action**	**Target**
Isoniazid	Inhibits mycolic acid synthesis and folate synthesis	Activated by KatG, targets InhA, KasA, and DfrA
Ethambutol	Inhibits arabinogalactan synthesis	Possibly EmbB
Ethionamide/prothionamide^*^	Inhibits mycolic acid synthesis	Activated by EthA, targets InhA
Cycloserine/terizidone^*^	Inhibits peptidoglycan synthesis by blocking the synthesis and use of D-alanine	Targets Alr and ddlA
Para-amino salicylic acid	Inhibits folate metabolism	DfrA
TMC207	Inhibits ATP synthase	AtpE
BTZ043	Inhibits essential cell-wall arabinan synthesis	DprE1
V-13-011503/V-13-012725	Inhibits cholesterol catabolism	HsaAB
V-13-009920	Inhibits the methylcitrate cycle	PrpC

* *Drugs with common targets have been grouped*.

### Calculating Drugged Metabolic States of Mtb With sMtb-RECON

After obtaining drug weight vectors, **c**_*d, i*_ for each drug, first, the bi-objective optimization problem as described in Rienksma et al. (2018), was solved:

$$w=\max\left\{\left(\sum_{i=1}^{n}-f_{e,i}\;\cdot \;\left|v_{i}\right|\right)+f_{b}\;\cdot \;v_{b}\right\}$$

subject to:

$$\begin{array}{l} S\;\cdot \;v\;=\;b\\ 1\leq v\leq u\text{,} \end{array}$$

wherein *w* is the objective function value, *v*_*i*_ represents the flux or rate of a reaction catalyzed by at least one enzyme; *f*_*e, i*_ represents the weight factor for each of those reactions; *v*_*b*_ represents the specific growth rate, i.e., the flux through the condition-specific biomass reaction; *f*_*b*_ represents the weight factor for the biomass reaction; *n* is the total number of reactions catalyzed by at least one enzyme; **S** represents the stoichiometric matrix; **v** represents a vector with all fluxes (comprising *v*_*i*_ and *v*_*b*_); **b** represents a vector with zeros; **l** represents a vector with lower bounds for all fluxes and **u** represents a vector with upper bounds for all fluxes. The lower bounds for the nutrients available to Mtb in model sMtb-RECON were set according to the condition-specific biomass reaction obtained for the macrophage part.

Afterwards, the bi-objective optimization problem is altered such that the objective function value that was obtained is set as a constraint. A new optimization problem is formulated to calculate the minimal flux through the reaction(s) affected by the respective drug:

$$w^{\prime }=\min\left\{c_{d,i}\;\cdot \;v\right\}$$

subject to:

$$\begin{array}{l} w=\left(\sum_{i=1}^{n}-f_{e,i}\;\cdot \;\left|v_{i}\right|\right)+f_{b}\;\cdot \;v_{b}\\ S\;\cdot \;v\;=\;b\\ 1\leq v\leq u\text{.} \end{array}$$

Wherein *w'* represents the new objective function value and **c**_*d, i*_ represents the drug weight vector for the current drug, *i*. Finally, a third optimization problem is formulated:

$$w=\max\left\{\left(\sum_{i=1}^{n}-f_{e,i}\;\cdot \;\left|v_{i}\right|\right)+f_{b}\;\cdot \;v_{b}\right\}$$

subject to:

$$\begin{array}{l} f_{d}\;\cdot \;w^{\prime }=\left\{c_{d,i}\;\cdot \;v\right\}\\ S\;\cdot \;v\;=\;b\\ 1\leq v\leq u\text{.} \end{array}$$

Wherein *f*_*d*_ is a value that is gradually lowered from 1 to 0 to represent increasing drug dosages, wherein a value of 1 represents no drug is applied or total ineffectiveness of the drug, and a value of 0 represents total effectiveness of the drug.

## Results

### Host-Pathogen Model sMtb-RECON

Model sMtb-RECON has a total of 8,987 reactions and 13.4% are from Mtb. Model sMtb-RECON contains 6,373 metabolites and 2,605 genes, of which 16.5 and 35.7% are from Mtb, respectively. RECON 2.2 has 9 compartments in total (number of metabolites indicated between brackets): cytoplasm (1,918), extracellular space (770), Golgi apparatus (312), lysosome (291), mitochondrion (756), nucleus (161), endoplasmic reticulum (675), peroxisome (440), and the mitochondrial intermembrane space (1). No phagosome compartment is available in RECON 2.2. There is a lysosome, but Mtb is known to block phagosome-lysosome fusion, therefore, the metabolites in this compartment are not likely to be available as nutrients for Mtb. Mtb is however assumed to acquire access to the cytosol. As this compartment contains the majority of the biomass precursors for the host, Mtb is assumed to have access to these. The cytosolic biomass precursors in model RECON 2.2 are thus set as metabolites that can be taken up by the Mtb part of model sMtb-RECON. A list of the metabolites and their maximal uptake rates is given in Additional File 1. Model sMtb-RECON is available in Additional File 2 and bounds used in the simulations are given in Additional File 3.

### Modeling Host-Pathogen Interaction

We extended the method presented in Rienksma et al. (2018) to integrate model and gene expression data to arrive at a model describing the metabolic state of the system during infection. The approach is summarized in Figure 1. We used dual RNA sequencing data obtained 24 h after exposing macrophage-like THP-1 cells to *Mycobacterium bovis* BCG, a close relative to Mtb (Rienksma et al., 2015).

First, the combined sMtb-RECON model was modified so that all reversible reactions of the Mtb part of sMtb-RECON were split in a forward and backward reaction, to make the sMtb part of the model irreversible (thus bringing the total number of reactions to 9,408). Then the combined model was constrained using the dual RNA seq data and condition-specific biomass reactions were obtained, for host and pathogen, by maximizing each human biomass precursor one-by-one for both the Mtb and the human part of sMtb-RECON. Afterwards, the constraints placed on sMtb-RECON were removed and the condition-specific biomass reaction of the human part of sMtb-RECON was used as a proxy for nutrient availability for the Mtb part of sMtb-RECON. The maximum allowable uptake rates of the Mtb part were thus limited to the maximum obtainable fluxes for each human biomass precursor. Thereafter, all reactions affected by a drug were gradually constrained as metabolism starts to reroute.

Using this method, uptake and secretion profiles can be predicted. This allows prediction of metabolites that are taken up or secreted even if in the starting model no additional constraints are imposed to limit uptake or secretion rates, apart from the oxygen uptake rate. Mtb encounters a hypoxic environment inside the host (Schnappinger et al., 2003; Matta and Kumar, 2016; Prosser et al., 2017). Therefore, we used the oxygen uptake rate to constrain the model to such an extent, that prediction of uptake and secretion rates becomes feasible without arbitrarily chosen limits on other uptake and/or secretion rates.

For the simulations of metabolic states using sMtb-RECON, three different assumptions have been made. The first being that the non-growth associated maintenance flux is 0.1 mmol g${{}_{\text{DW}}}^{-1}$ h^−1^ or higher, as this maintenance flux was shown to have the best fit to experimental data (Rienksma et al., 2014), the second being that the condition-specific biomass reaction is maximized and the third being that overall enzyme usage is minimized. The first requirement is set as a constraint, while requirements two and three are captured as a bi-objective function in a bi-objective optimization problem. The weight factor ratio, *f*_*r*_, between the condition-specific biomass reaction weight, *f*_*b*_, and the total enzymatically catalyzed reaction weight, *f*_*e*_, equals 0.8. By lowering this factor, more emphasis is put on the minimization of enzyme usage, while increasing this factor puts more emphasis on attaining a higher value for the condition-specific biomass reaction. Lowering this factor would result in a larger part of nutrients being used by Mtb to generate energy, while increasing this factor would result in the uptake profile looking more similar to the condition-specific biomass reaction itself. Lowering and raising *f*_*r*_ is however limited to a range wherein a single objective of the bi-objective optimization problem is not dominant over the other (Rienksma et al., 2018).

### Metabolic State During Infection

**Figure 2:**
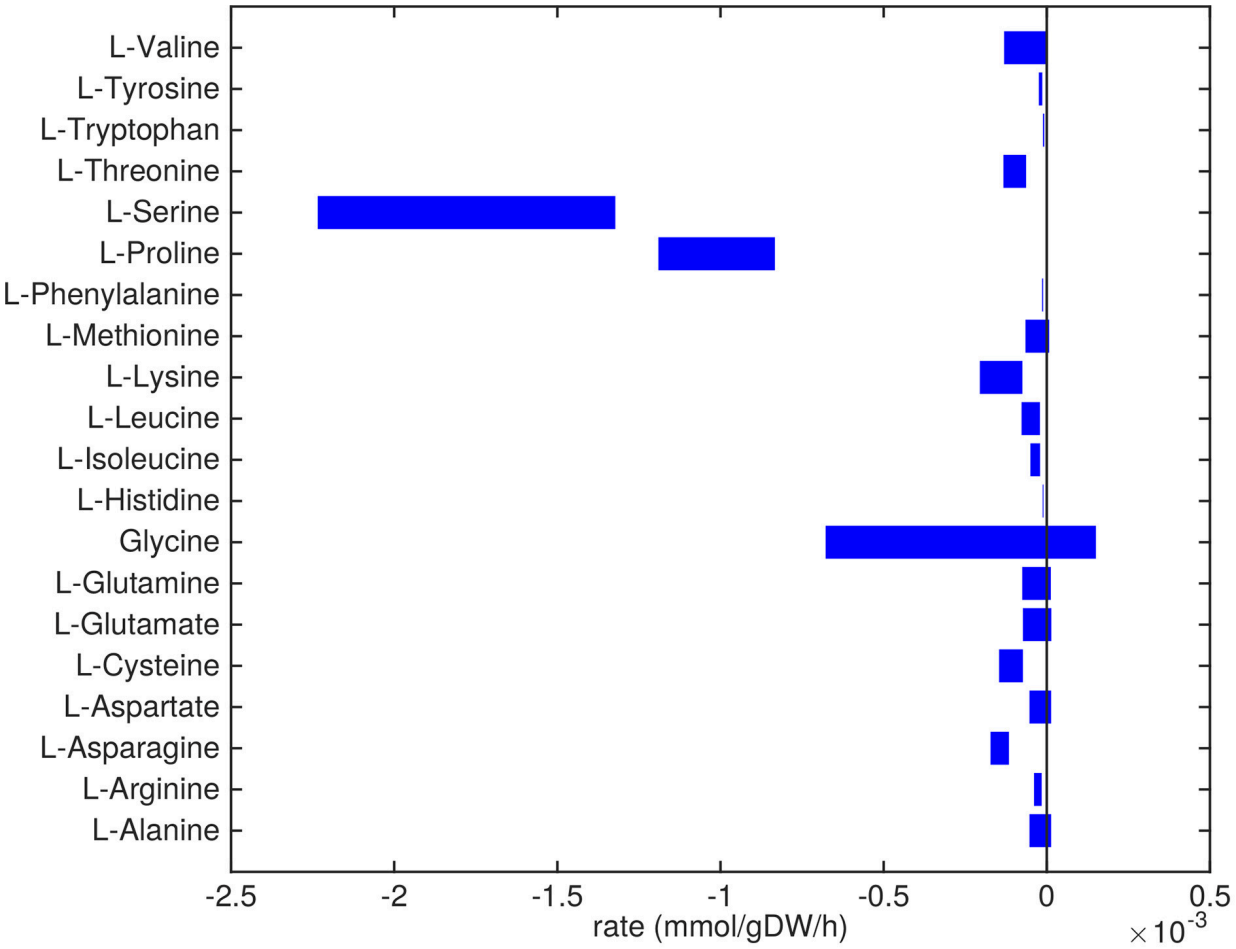
Predicted amino acids uptake and secretion rates by Mtb in the host. Predicted ranges of uptake and secretion rates (mmol g${{}_{\text{DW}}}^{-1}$ h^−1^) of amino acids by Mtb inside the host are indicated by blue bars. Negative values denote uptake and positive values denote secretion.

**Figure 3:**
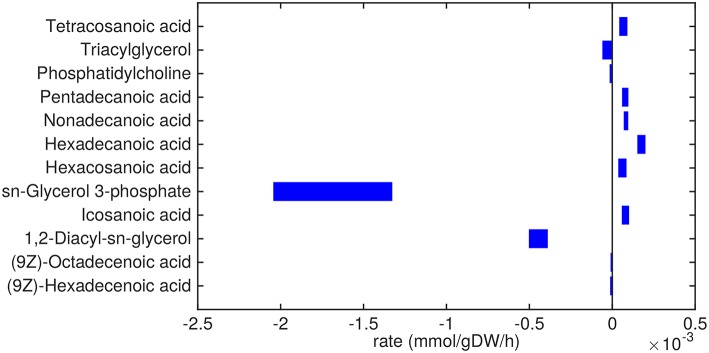
Predicted lipid uptake and secretion rates by Mtb in the host. Predicted ranges of uptake and secretion rates (mmol g${{}_{\text{DW}}}^{-1}$ h^−1^) of lipids by Mtb inside the host are indicated by blue bars. Negative values denote uptake and positive values denote secretion.

**Figure 4:**
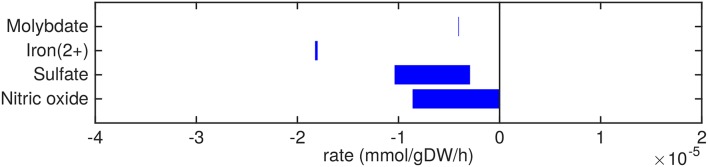
Predicted uptake and secretion rates of cofactors and small molecules by Mtb in the host. Predicted ranges of uptake and secretion rates (mmol g${{}_{\text{DW}}}^{-1}$ h^−1^) of cofactors and small molecules by Mtb inside the host are indicated by blue bars. Negative values denote uptake and positive values denote secretion.

**Figure 5:**
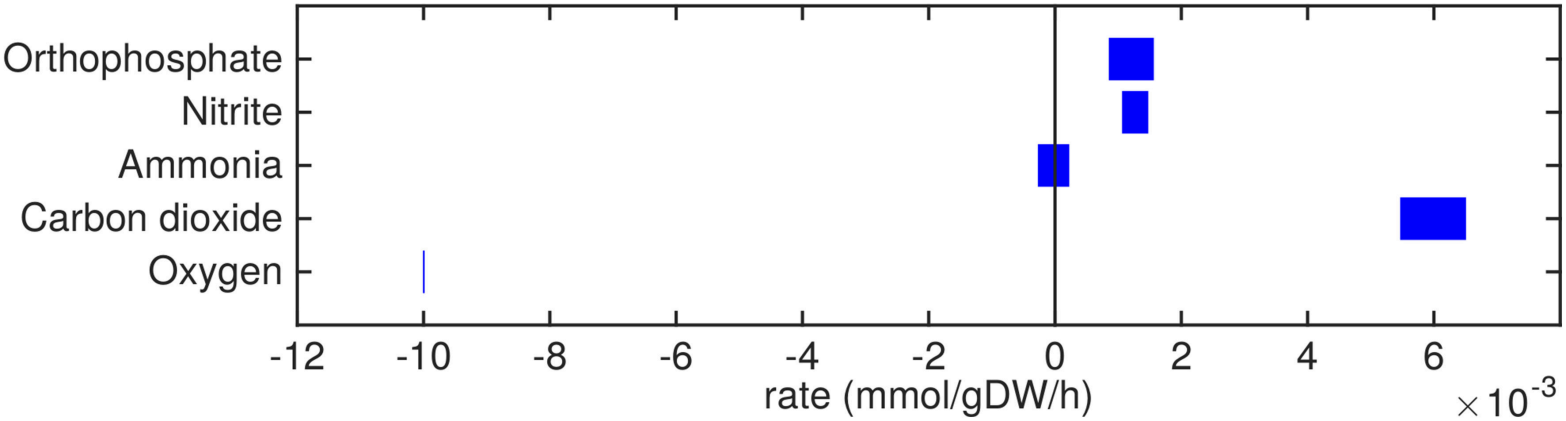
Predicted uptake and secretion rates of molecular oxygen, carbon dioxide and other small molecules by Mtb in the host. Predicted ranges of uptake and secretion rates (mmol g${{}_{\text{DW}}}^{-1}$ h^−1^) of oxygen, carbon dioxide, and other small molecules by Mtb inside the host are indicated by blue bars. Negative values denote uptake and positive values denote secretion.

Mtb is predicted to take up and secrete a plethora of different metabolites at varying rates (Figures 2–**5**). A comprehensive list of predicted uptake and secretion rates is given in Additional File 1.

**Figure 6:**
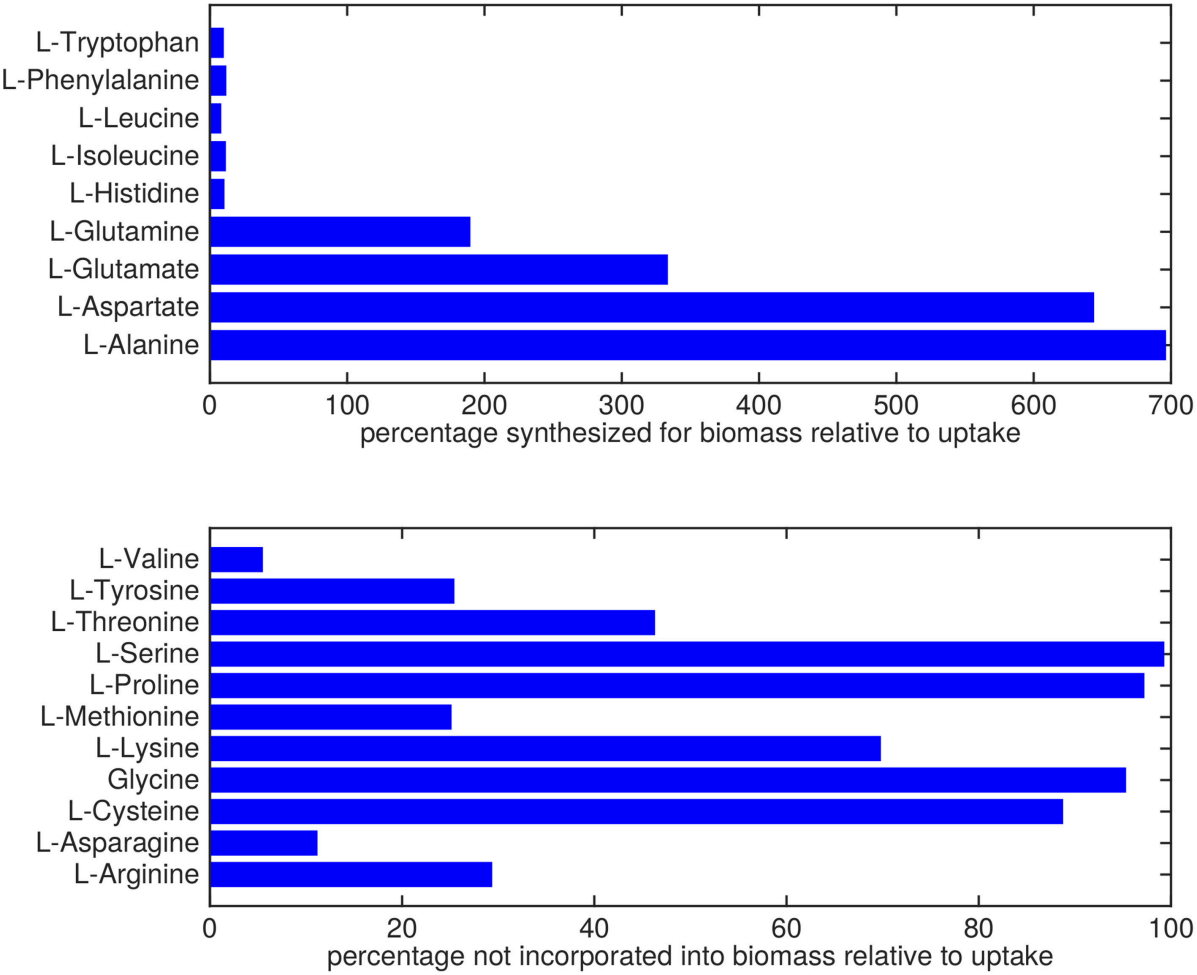
Usage of amino acids derived from the host. Top: Rate of amino acids synthesis by Mtb for biomass incorporation relative to their respective uptake rates. Bottom: Relative rate of amino acid uptake that is used in processes other than biomass synthesis.

Almost all amino acids are predicted to be taken up by Mtb (Figure 2). Most notably serine and proline are predicted to be taken up at relatively high rates. Glycine uptake and/or secretion rates remain largely underdetermined, in fact using this approach the model is not able to predict whether it is produced or consumed. Notably, our approach also allows a small production of glutamine, glutamate, aspartate and alanine. Glutamate can be interconverted to glutamine by, for example, glutamine synthase at the expense of ATP (Tullius et al., 2003). In this way any additional uptake of glutamate can serve as a potential source of glutamine, or the other way around, although ATP expenditure limits this interconversion. Therefore, the ranges wherein glutamate and glutamine are predicted to be taken up are equal. The pattern observed in Figure 2 is not a reflection of the coefficients for the amino acids in the condition-specific biomass reaction of sMtb, as one would perhaps expect. This can be seen in Figure 6, where one would expect that all amino acids that are taken up, would be incorporated into biomass, which is obviously not the case. By multiplying the flux through the condition-specific biomass reaction with the respective column of the stoichiometric matrix corresponding to this biomass reaction, the fluxes required for synthesis of the individual biomass precursors can be obtained. When comparing the fluxes required for biomass synthesis with their respective predicted uptake rates, most notably alanine and aspartate are predicted to be synthesized by Mtb (Figure 6, upper panel). On the other hand, almost all serine, proline and glycine is used for purposes other than biomass synthesis, i.e., ATP and NADH production required for maintenance (Figure 6, lower panel). Such behavior has been described in cancer cells (Tedeschi et al., 2013; Amelio et al., 2014), but not for Mtb.

Glycerol-3-phosphate is a lipid (precursor) that is taken up in a relatively high amount (Figure 3). It is known that glycerol-3-phosphate serves as a major carbon source for several intracellular pathogens (Eisenreich et al., 2010) and it has been suggested that glycerol-3-phosphate might serve as an alternative carbon source for Mtb *in vivo* (Pieters and McKinney, 2013).

Glycerolipids such as diacylglycerol (DAG), triacylglycerol (TAG), and phosphatidylcholine are predicted to be taken up as well. Most notably, DAG is taken up in relatively large amounts (0.5 mmol g${{}_{\text{DW}}}^{-1}$ h^−1^). These three metabolites are closely related, as phosphatidylcholine can be converted to DAG and choline phosphate by phospholipase C (Bopape et al., 2004). DAG can be converted to TAG, which is subsequently stored in lipid droplets (Daniel et al., 2011). Unsaturated fatty acids (octadecenoic and hexadecenoic acids) are predicted to also be taken up. On the other hand, a range of saturated fatty acids are predicted to be secreted. These fatty acids are derived from TAG and DAG, indicating that there is a higher requirement for the glycerol backbone of TAG and DAG than for the attached fatty acids.

Small metal cofactors, such as molybdate and iron are predicted to be taken up (Figure 4). Mtb is known to chelate iron using siderophores, called mycobactins, via a specialized ESX-3 system (Siegrist et al., 2009). This ESX-3 system is essential for *in vitro* growth (Griffin et al., 2011). In sMtb, iron as an ion or element, without being integrated in a larger molecule, is not incorporated in the condition-specific biomass reaction, as is the case in most biomass reactions of models of Mtb metabolism (Jamshidi and Palsson, 2007; Rienksma et al., 2014). However, iron incorporated in larger molecules, such as heme groups, is present in the condition-specific biomass reaction(s) of sMtb.

The excretion of orthophosphate and nitrite (Figure 5) are probably artifacts from the model, where phosphate might be derived from the phosphate group of glycerol-3-phosphate and nitrite could be related to the nitrogen groups of the variety of amino acids that are taken up. The model predictions show that free ammonia can be taken up as well as secreted at about equal rates, so the fate of ammonia uptake or secretion remains inconclusive from these predictions.

The oxygen uptake rate equals 0.01 mmol g${{}_{\text{DW}}}^{-1}$ h^−1^ which equals the imposed maximum uptake rate (Figure 5). Decreasing the lower bound on oxygen exchange, i.e., allowing a higher uptake rate of oxygen, results in a higher specific growth rate. As such, the system is limited by oxygen and it is obvious that oxygen is taken up at its maximum rate. Carbon dioxide is secreted mainly due to respiration.

### Metabolic States of Drugged Mtb: Rerouting Metabolism

Table 1 illustrates the mode of action of 12 anti-TB drugs known to interfere with metabolic enzyme activity with known targets. To simulate increasing dosages of these drugs, we gradually decreased the flux through the reactions catalyzed by the affected enzymes.

**Figure 7:**
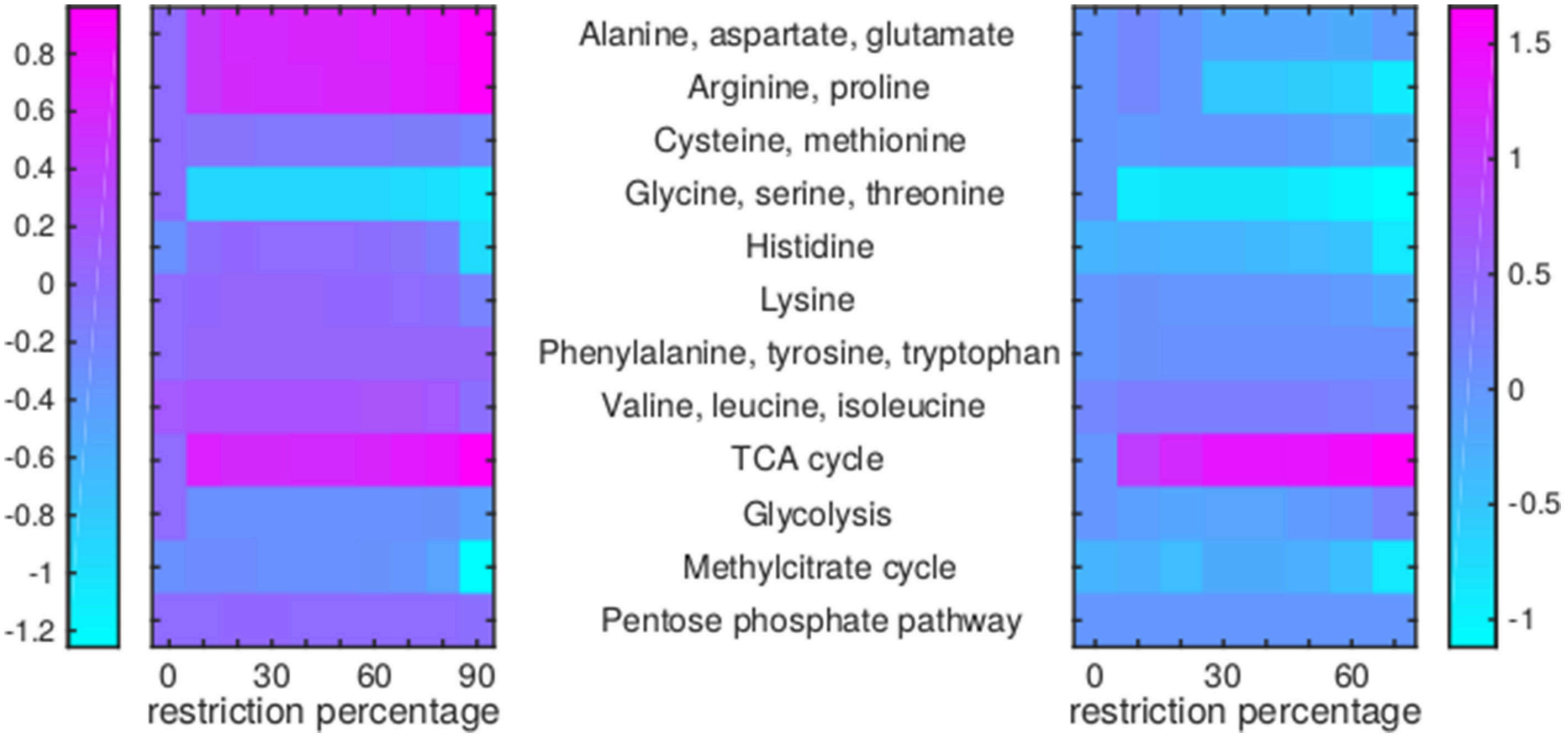
Flux rerouting through amino acid metabolism and major pathways upon application of drugs. Heat maps indicate a predicted relative increase (pink) or decrease (light blue) through various pathways upon the application of a variety of drugs. The logarithm of the sum of all absolute fluxes is given per pathway (values are indicated in the color bars on either side), so information on directionality is not comprised. The x-axis indicates the percentage of restriction of the drug-affected reaction(s). Left: average of BTZ043, cycloserine/terizidone, ethambutol, ethionamide/propionamide, and isoniazid; Right: TMC207.

**Figure 8:**
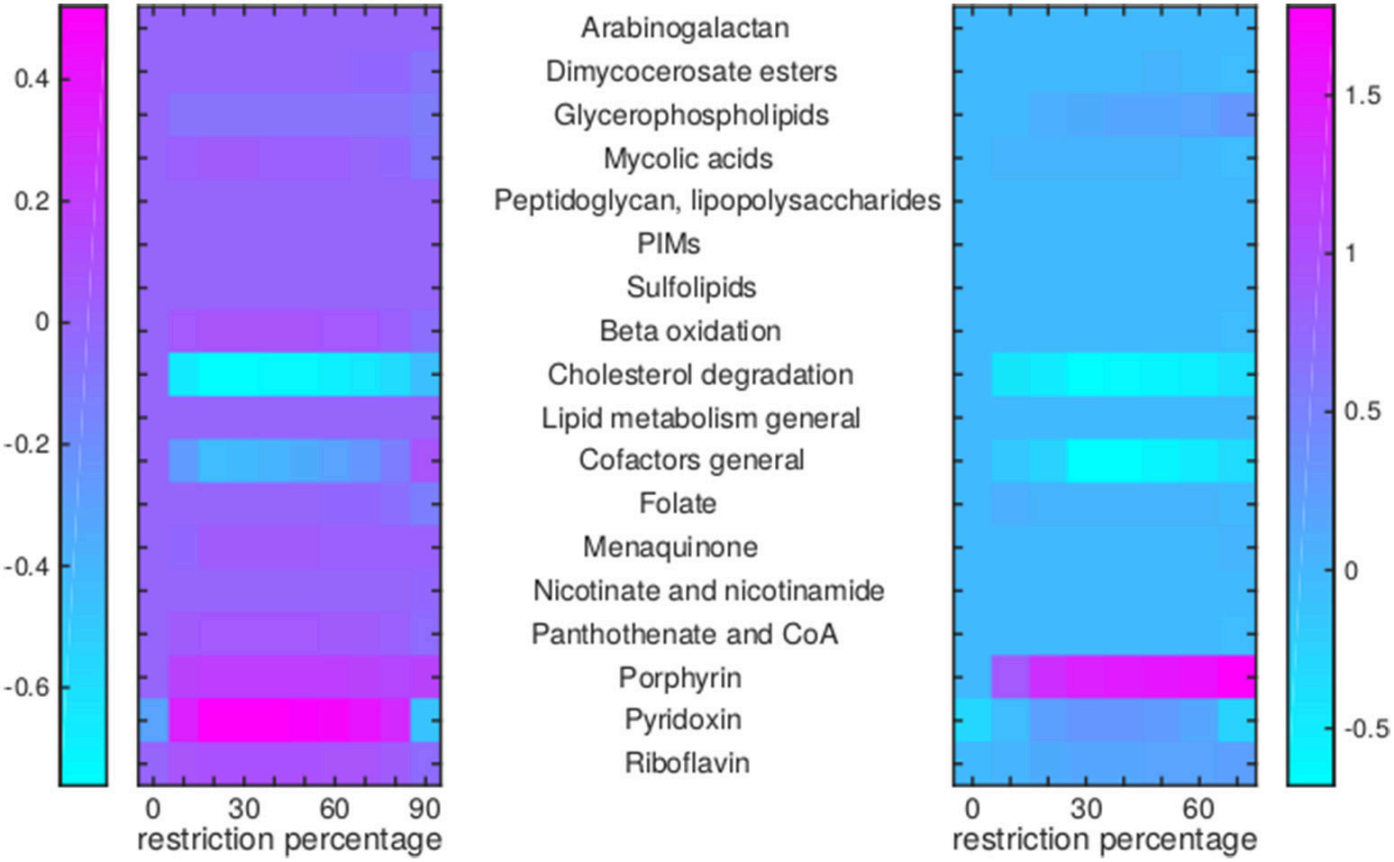
Flux rerouting through cell wall component metabolism, lipid metabolism, and cofactor metabolism upon application of drugs. Heat maps indicate a predicted relative increase (pink) or decrease (light blue) through various pathways upon the application of a variety of drugs. The logarithm of the sum of all absolute fluxes is given per pathway (values are indicated in the color bars on either side), so information on directionality is not comprised. The x-axis indicates the percentage of restriction of the drug-affected reaction(s). Left: average of BTZ043, cycloserine/terizidone, ethambutol, ethionamide/propionamide, and isoniazid; Right: TMC207.

**Figure 9:**
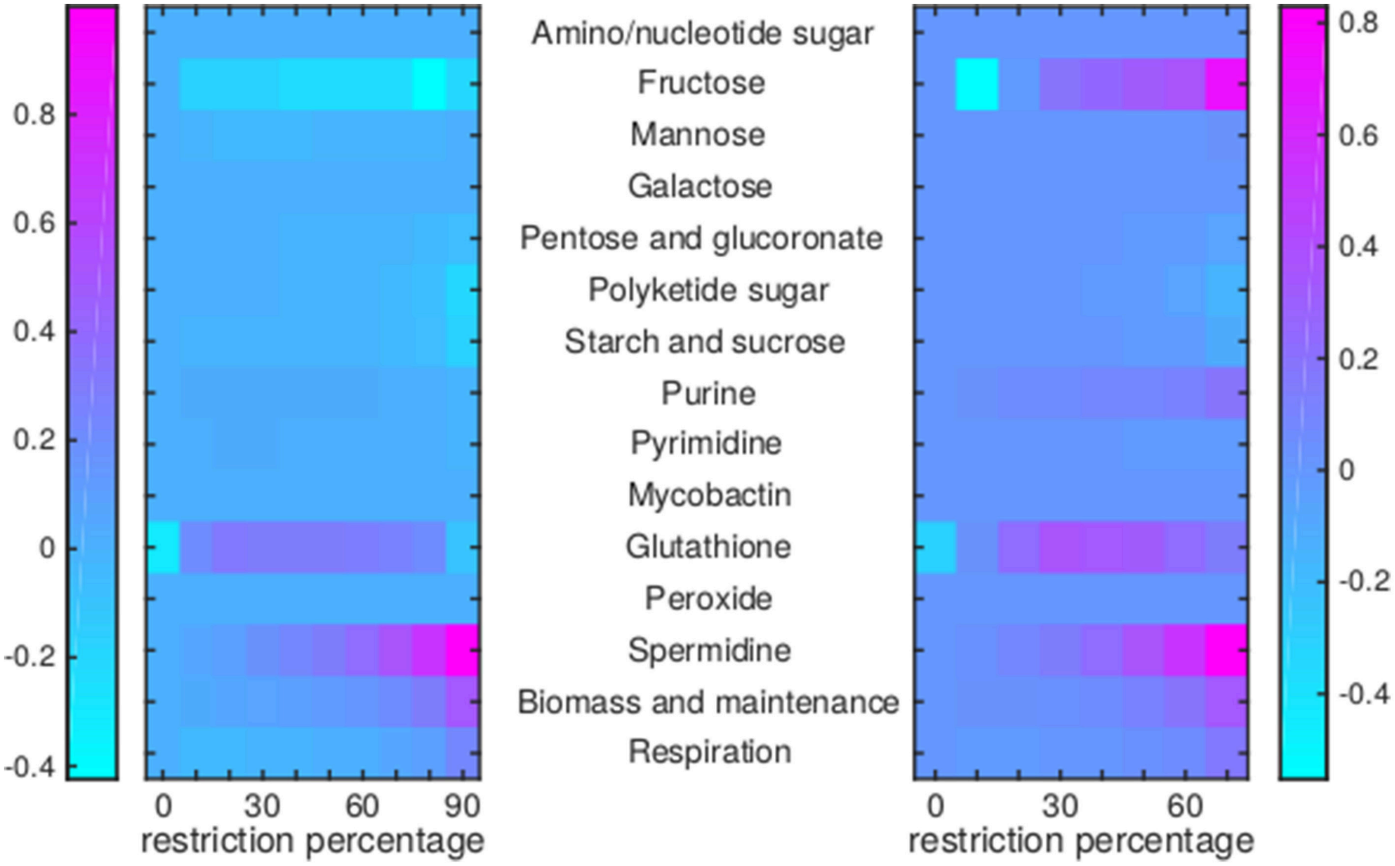
Flux rerouting through sugar metabolism, nucleotide metabolism, and various other metabolic pathways upon application of drugs. Heat maps indicate a predicted relative increase (pink) or decrease (light blue) through various pathways upon the application of a variety of drugs. The logarithm of the sum of all absolute fluxes is given per pathway (values are indicated in the color bars on either side), so information on directionality is not comprised. The x-axis indicates the percentage of restriction of the drug-affected reaction(s). Left: average of BTZ043, cycloserine/terizidone, ethambutol, ethionamide/propionamide, and isoniazid; Right: TMC207.

Of the 12 drugs in Table 1, some are grouped as they have the same enzyme target, resulting in nine drugs or groups of drugs with different targets. No effect on Mtb metabolism could be predicted for three of these: V-13-011503/V-13-012725, V-13-009920, and para-amino salicylic acid. The drugs BTZ043, cycloserine/terizidone, ethambutol, ethionamide/propionamide, and isoniazid are predicted to have a very similar effect on metabolism and their effect is therefore grouped (Figures 7–9, left panels), even though their enzymatic targets are very different (Table 1). Notably, TMC207 has a very different effect on metabolism (Figures 7–**9**, right panels).

For TMC207, the flux through alanine, aspartate, glutamate, arginine, and proline metabolism becomes relatively low (Figure 7), while the flux through porphyrin metabolism and fructose metabolism becomes relatively high, as the percentage of constriction of the reactions affected by TMC207 increases (Figures 8, 9). Porphyrins are heterocyclic compounds able to form metal complexes, such as heme, the latter attenuating growth of Mtb if absent from the growth medium (Owens et al., 2013). In addition, with moderate constriction (around 40% in Figures 8, 9) flux is lowered through pathways such as cholesterol degradation and cofactor metabolism in general while flux trough these pathways is relatively large when the drug-affected reactions are constrained mildly (< 10%) or heavily (>70%). Metabolism of glutathione, an antioxidant, shows behavior opposite to that of cholesterol degradation and cofactor metabolism in general (Figure 9).

**Figure 10:**
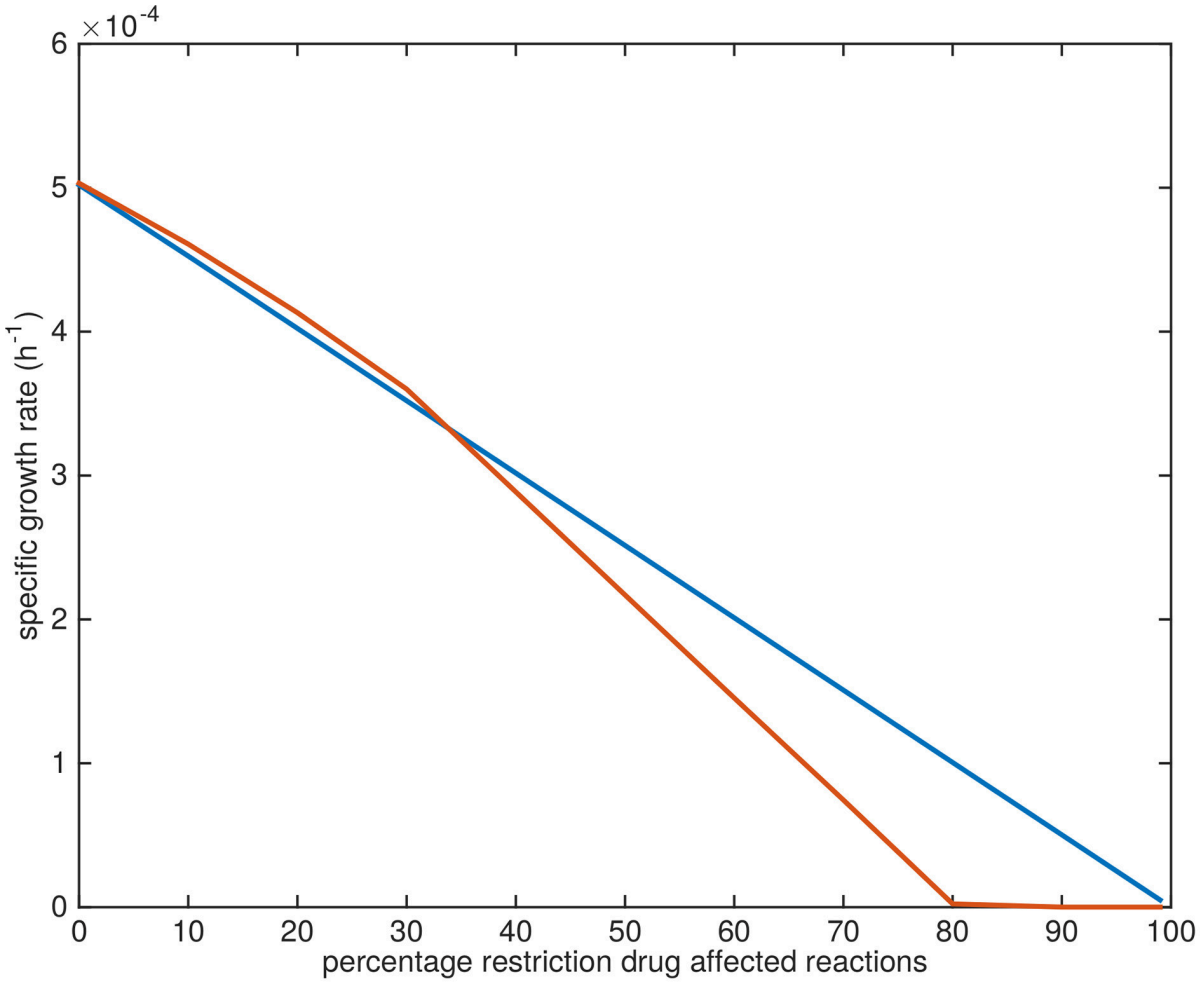
Growth rate decline upon application of drugs. Blue line: predicted average specific growth rate of Mtb upon application of BTZ043, cycloserine/terizidone, ethambutol, ethionamide/propionamide, or isoniazid. Red line: predicted average specific growth rate of Mtb upon application of TMC207.

The specific growth rates gradually approach zero upon the application of the drugs. The application of TMC207 is predicted to result in a relatively faster drop to zero growth rate (at 80% restriction of the flux through the affected reactions instead of 100%) (Figure 10).

## Discussion

Mtb is under considerable stress from the host during infection. This situation can worsen with the application of drugs. As the dosage of a metabolic drug(s) increases, so does the pressure on Mtb to circumvent the effects of the(se) drug(s) by rerouting metabolism. The percentage of restriction, as shown in Figures 7–10 can be viewed as a proxy for the drug dosage, as the effectiveness of a drug is dependent on its ability to interact with as many target enzymes as possible. As metabolism is an interconnected network, alternative metabolic states can exist to bypass the part of Mtb metabolism that is malfunctioning due to the effect of the drug(s).

The host-pathogen model first created by Bordbar et al. (2010), iAB-AMØ-1410-Mt-661, contained 2,071 genes associated to 4,490 reactions describing interconversions between 3,399 metabolites. The sMtb-RECON model here presented represents a major improvement in scope. The number of reactions and metabolites has almost doubled (8,987 reactions and 6,373 metabolites in sMtb-RECON) whereas there has been a 25% increase in the number of associated genes (2,605). The increase in reactions and metabolites is mainly driven by the expanded coverage of RECON 2.2 (Thiele et al., 2013) compared to RECON 1 (Duarte et al., 2007). RECON 1 accounts for 2,766 metabolites and 3,311 metabolic reactions whereas RECON.2.2 describes 5,324 metabolites and 7,785 reactions. In addition to the expanded scope, reactions in RECON 2.2 are mass and charge balanced, which is not always the case in RECON 1. This curation effort rendered RECON 2.2 able to correctly predict ATP yield on different carbon sources. Even though, the generation of iAB-AMØ-1410-Mt-661 entailed a manual curation step some of these unbalanced reactions can still be found in this model, which limits its predictive value regarding energy generation by the host.

Both RECON 1 and RECON 2.2 describe a relatively similar number of genes 1,496 and 1,675, respectively for. The increase in the number of genes in sMtb-RECON compared with iAB-AMØ-1410-Mt-661 is mainly due to the more detailed description of metabolism of Mtb provided by sMtb compared to iNJ661 (on which the iAB-AMØ-1410-Mt-661 model was based). Model sMtb was seen to outperform previously published models, among which iJN661, regarding predictions on gene essentiality, metabolic state, and drug associated phenotype. Additionally, sMtb includes pathways, such as the cholesterol degradation pathway, specifically relevant for intracellular growth and survival. sMtb-RECON inherits the respective improvements of sMtb and RECON 2.2.

Both sMtb and iAB-AMØ-1410-Mt-661 where specifically designed to model *in vivo* conditions of Mtb and in both cases, the biomass reaction was modified through incorporation of gene expression data. However, important differences appear. In the case of AB-AMØ-1410-Mt-661, the microarray data described gene expression levels in the host (Thuong et al., 2008), whereas the dual RNA seq data used in this study captures the transcriptional response of both the host and the pathogen.

Data used in this study were obtained for *M. bovis* BCG 24 h after infection of the human macrophage-like cell line THP-1. Mtb and *M. bovis* BCG are closely related, however differences between both renders Mtb a human pathogen and *M. bovis* BCG a vaccine strain (O'Reilly and Daborn, 1995). When tuberculosis as a human disease is studied, it is obvious that *M. bovis* BCG is not suited as a study object. However, here metabolism is modeled to ultimately identify response to drugs that can lead to the development of new drugs or therapeutic strategies. Even though many of the differences between both species pertain metabolic capabilities (Rehren et al., 2007), when the entire metabolism of both species is compared, only minor differences appear (Lofthouse et al., 2013). So, if a metabolic drug is found that can eradicate *M. bovis* BCG, it is likely that it can eradicate Mtb as well. There are however obvious differences between Mtb and *M. bovis* BCG and some having an impact on metabolism are discussed below.

*M. bovis* BCG has a specific point mutation of a C to G in position 169 of the *pncA* gene encoding pyrazinamidase (Scorpio et al., 1997). This mutation results in a loss of activity of the pyrazinamidase. The effective first line drug pyrazinamide (PZA) is a prodrug that is converted to its active form by this pyrazinamidase. The loss of activity thus has severe implications, as mycobacteria having this point mutation are resistant to PZA (Juréen et al., 2008). Another difference between *M. bovis* BCG and Mtb lies within their regulation of cholesterol degradation. Cytochrome P450s Cyp125 and Cyp142 are encoded in the genomes of both *M. bovis* BCG and Mtb. These cytochrome P450s catalyze the first steps of the degradation of the side chain of cholest-4-en-3-one (Ouellet et al., 2011). In *M. bovis* BCG *cyp142* contains a specific point mutation, resulting in a premature stop codon and a non-functional product. There is thus redundancy and thus backup of cholesterol oxidation capacity in Mtb as compared to *M. bovis* BCG (Driscoll et al., 2010). In addition to these point mutations altering the function of very specific genes, there are whole genes present in the genome of Mtb, while absent in the genome of *M. bovis* BCG, and vice versa. One such example is the type VII ESX secretion systems. *M. bovis* BCG lacks the ESX-1 secretion system and ESX-1 secreted proteins EsxA (ESAT-6) and EsxB (CFP-10) (Mahairas et al., 1996; Gey van Pittius et al., 2001; Gröschel et al., 2016). There is increasing evidence that this system is used to access the cytosol already during the early stages of infection (Manzanillo et al., 2012; Simeone et al., 2012; Augenstreich et al., 2017). The absence of these genes perhaps causes a difference in nutrient availability for Mtb and *M. bovis* BCG. The ESX-1 secretion system and secreted protein of Mtb could provide access to a whole range of nutrients that *M. bovis* BCG under the same conditions does not have access to. This would imply that the metabolic states of both bacteria could vary significantly under intra-host conditions. Dual RNA sequencing performed on Mtb (Zimmermann et al., 2017) might therefore result in an improved modeling of the intracellular state of Mtb, however currently available data only represent the very early response (4 h post infection).

Similarly, data from the human macrophage-like cell line THP-1 have been used to characterize the host. These cells were induced to differentiate into mature macrophages and they have been shown to behave, after infection, in a manner similar to that of monocyte-derived macrophages (Riendeau and Kornfeld, 2003). They have been shown to exhibit similar responses regarding receptor expression, bacterial uptake, survival, and drug response (Stokes and Doxsee, 1999). Similarities also appear regarding metabolic responses, such as levels oxygen radicals and lack of nitric oxide production (Sly et al., 2001; Fontán et al., 2008), however we cannot exclude other differences in metabolism.

For the simulations of metabolic states using sMtb-RECON we have considered biomass maximization and minimization of overall enzyme utilization using a bi-objective optimization approach. However, this does not result in the direct uptake of all amino acids in a ratio that is proportional to the ratio of the corresponding coefficients in the condition-specific biomass reaction (Figure 6). As the direct uptake of amino acids represents a much shorter, and thus less enzyme intensive route, such a route would be preferred if the only function of the amino acids were direct incorporation into biomass. This is however not the case, because the amino acids are also used to generate energy in the form of ATP. Whether amino acids are required to synthesize biomass or are required to generate energy or both, it has been shown that Mtb is reliant on amino acids to thrive within the host (Gouzy et al., 2013, 2014a,b).

Some nutrients are closely related and their interconversion involves the usage of one or only a few enzymes. These interlinked metabolites, such as glutamate/glutamine and TAG/DAG/phosphatidylcholine can be relatively easily substituted and predictions on their individual uptake and/or secretion rates can only be derived from the predictions of their combined uptake and/or secretion rate. This is especially visible in Figure 2, where the ranges of uptake and secretion rates are equal for both glutamate and glutamine.

Perhaps surprisingly, the metabolic states predicted with sMtb-RECON after perturbation with BTZ043, cycloserine/terizidone, ethambutol, ethionamide/propionamide, or isoniazid are all highly similar. For some drugs, such as isoniazid and ethionamide/propionamide, this can be explained by an overlapping enzyme target InhA. Inhibition of this enoyl acyl carrier protein (ACP) reductase is one of the most effective ways to eradicate Mtb. This enzyme catalyzes 2-trans-enoyl ACP reduction and catalyzes the final step in fatty acid synthesis and is involved in mycolic acid synthesis. Inactivation of InhA results in cell wall alterations and eventually lysis of the cell (Duan et al., 2014). However, the mycolic acid synthesis inhibition caused by isoniazid and ethionamide/propionamide is not directly related to the cell wall synthesis inhibiting effects of ethambutol and cycloserine/terizidone. Even though there is evidence that arabinogalactan and mycolic acids are physically attached to each other, this is not reflected in model sMtb-RECON (Birch et al., 2008).

Nevertheless, both mycolic acids and arabinogalactan are part of the condition-specific biomass reaction of Mtb. The condition-specific biomass reaction of Mtb is based on RNA sequencing data derived from *M. bovis* BCG 24 h post infection (Rienksma et al., 2015). The ratio between the metabolic precursors in this biomass reaction is constant within model sMtb-RECON. Therefore, a decrease in the ability to synthesize mycolic acids, which are part of the biomass reaction, by constraining the reactions catalyzed by InhA, results in a decreased maximally achievable value of the condition-specific biomass reaction. This in turn will result in a decreased amount of arabinogalactan needed to achieve this value. The minimization of enzyme usage ensures that the overall flux through the arabinogalactan synthesis pathway is minimized. This process works the other way around as well. A limitation of the flux through the arabinosyltransferase EmbB, that is required for the synthesis of arabinogalactan (Goude et al., 2009), will result in a lower value of the condition-specific biomass reaction, in turn leading to a lower need of mycolic acids. The mycolic acid synthesis pathway is a highly linear pathway, and completely unidirectional in model sMtb-RECON. No ATP for maintenance can be generated by mycolic synthesis in the model. The lower need of mycolic acids will result in less flux through the mycolic acid synthesis pathway.

The reason the predicted metabolic state of TMC207-affected Mtb differs from the other drugs is due to the function of AtpE as an ATP generating enzyme involved in respiration. ATP is on the one hand a direct biomass precursor in the condition-specific biomass reaction, but it is also required to synthesize almost all other biomass precursors. In addition, ATP is required to satisfy the non-growth associated maintenance constraint. As such, the effect of constraining AtpE is not nearly as straightforward as the effect of constraining InhA or EmbB. This effect can be seen in Figure 10, wherein the effect of limiting AtpE, due to the application of TMC207, on the maximum condition-specific biomass reaction value is visible (red line). The line contains multiple bends, the most notable at 30%, but at 10 and 20% as well. These bends represent metabolic rerouting that can vary with the severity of constraining the respective reaction(s). An example of such variance can be seen in Figure 7, in the methyl citrate cycle. The flux through this cycle does not linearly increase or decrease with the constraining percentage at all, which can be more clearly seen when comparing the methyl citrate cycle in Figure 7 with a linear increase in biomass and maintenance, as seen in Figure 9. As TMC207 is predicted to have an effect that substantially differs from the effect of BTZ043, cycloserine/terizidone, ethambutol, ethionamide/propionamide, and isoniazid, a combination of TMC207 and the latter drugs would probably provide a more effective strategy to combat TB than combinations of drugs without TMC207.

Model RECON 2.2 is a general model of human metabolism. A macrophage is however, a very specialized human phagocytic cell, which engulfs and digests pathogens in a specialized compartment, the phagosome. An important mechanism of pathogen killing by phagocytes involves generating the superoxide anion, which reacts with iron sulfur clusters in the pathogen, releasing iron and subsequently damaging DNA (Hurst, 2012; Winterbourn and Kettle, 2012). RECON 2.2 does not have a phagosomal compartment and the applied metabolic state simulation strategy (Figure 1) assumes that all cytoplasmic biomass precursors for the macrophage are available for Mtb inside the phagosome, while the effect of or presence of other compounds is overlooked. The effect of oxygen radicals and resulting hydrogen peroxide is not captured by the approach applied in this study, which can be seen in Figure 9 where no change is visible in peroxide (degradation). The overall flux through peroxide degradation processes should increase relative to the flux through the condition-specific biomass reaction, assuming a more or less constant supply of superoxide anions by the macrophage.

Divalent metal cofactors such as iron, manganese, and zinc are essential for Mtb virulence (Zondervan et al., 2018). Currently, only the iron requirement is reflected in model sMtb-RECON in the form of heme being an essential precursor for Mtb biomass. The metal availability in cells in general is limited and proteins compete for these metals (Foster et al., 2014). Therefore, a better strategy would be to identify Mtb enzymes that require a certain metal cofactor and to simulate low availability of such a cofactor by constraining the total flux of all reactions associated with these enzymes (Wegrzyn et al., 2019). This could provide a more accurate representation of the metabolic state of Mtb during infection, especially as macrophages are known to use high affinity iron binding proteins to limit the availability of iron (Kurthkoti et al., 2017), making this a promising modeling strategy.

The question remains whether these predictions are accurate enough to warrant pinpointing specific genes and their corresponding enzymes as drug targets. Previous modeling efforts have shown a poor predictive power of essential genes, using a bi-objective optimization strategy (Rienksma et al., 2018). Continuous step-by-step improvements of Mtb models to reach one functional standardized model of Mtb metabolism is a solid step in this direction (Kavvas et al., 2018).

Understanding the metabolic rerouting upon drug administration can lead to the identification of new metabolic bottlenecks, the identification of new targets and in the long run the development of new therapies based on combination of drugs. Moreover, detailed analysis of the mechanisms deployed by Mtb to counteract the impact of drugs might offer insights on the role of genetic modifications related to the development of drug resistances.

## Author Contributions

RR drafted the manuscript, conceived the study, and performed the data analysis. MS-D participated in drafting the manuscript and helped with the data analysis. PS and VM participated in drafting the manuscript. All authors contributed to the study design and critically read, revised, and approved the manuscript.

### Conflict of Interest Statement

VM is the majority owner and managing director of Lifeglimmer GmbH. All other authors declare no conflict of interest.
