# Supplementary material for: Modeling Host-Pathogen Interaction to Elucidate the Metabolic Drug Response of Intracellular Mycobacterium tuberculosis
Source: Front Cell Infect Microbiol. 2019 May 8;9:144. doi: 10.3389/fcimb.2019.00144 (PMC6519342; doi:10.3389/fcimb.2019.00144)
Supplement: Supplementary file 1 [file Data_Sheet_1.pdf]

frontiers

Supplementary Material

Additional file 1 List of metabolites predicted to be available for Mtb inside the host and their predicted maximal uptake rates.

	Metabolite		Maximal uptake rate
	RECON 2	sMtb	(mmol·gDW^{-1}·h^{-1})
1	ala_L	ALA	1
2	pa_hs		1
3	amp	AMP	1
4	arg_L	ARG	1
5	asn_L	ASN	1
6	asp_L	ASP	1
7	atp	ATP	1
8	chsterol	CHOLESTEROL	0
9	cmp	CMP	0.37
10	cys_L	CYS	1
11	dag_hs	DAG	1
12	damp	DAMP	0.30
13	dcmp	DCMP	1
14	dgmp	DGMP	0.28
15	dtmp	DTMP	0.08
16	gln_L	GLN	1
17	glu_L	GLU	1
18	gly	GLY	0.02
19	glygn2		0.21

20	gmp	GMP	1
21	hdca	HEXADECANOATE	0.32
22	hdcea	9HEXADECENOATE	1
23	his_L	HIS	1
24	ile_L	ILE	1
25	leu_L	LEU	1
26	met_L	MET	0.12
27	ocdca	OCTADECANOATE	1
28	ocdcea	9OCTADECENOATE	0.03
29	pail_hs	PITBA	0.23
30	pchol_hs	PHOSPHATIDYLCHOLINE	1
31	pe_hs	ETHA	1
32	pglyc_hs	PG	1
33	phe_L	PHE	1
34	pro_L	PRO	1
35	ps_hs	PS	1
36	ser_L	SER	0
37	sphmyln_hs		0.05
38	tag_hs	TAG	1
39	thr_L	THR	1
40	trp_L	TRP	0
41	ttdca	TETRADECANOATE	1
42	tyr_L	TYR	1
43	ump	UMP	1
44	val_L	VAL	1
45	adp	ADP	1
46	no	NO	1
47	co2	CO2	1
48	glyc3p	GL3P	1
49	o2	O2	1

50	lys_L	LYS	1

Additional file 2 model sMtb-RECON in xml format

Additional file 3 Bounds for sMtb-RECON used in the simulations.
